# A Multi-Level Approach to Childhood Obesity Prevention and Management: Lessons from Japan and the United States

**DOI:** 10.3390/nu17050838

**Published:** 2025-02-28

**Authors:** Qutaibah Oudat, Sarah E. Messiah, Alia Dawlat Ghoneum

**Affiliations:** 1 Department of Population Health, College of Nursing, University of Cincinnati, Cincinnati, OH 45221, USA; 2 Peter O’Donnell Jr. School of Public Health, University of Texas Southwestern Medical Center, Dallas, TX 75390-8876, USA; sarah.messiah@utsouthwestern.edu; 3 Department of Family Medicine, East Carolina University, 101 Heart Drive, Greenville, NC 27834, USA; ghoneuma23@ecu.edu

**Keywords:** childhood obesity, public health policies, cultural dietary habits, lifestyle factors, Shokuiku, National School Lunch Program, obesity prevention, Japan, United States

## Abstract

Background: Childhood obesity is a pressing global public health challenge, marked by significant disparities in prevalence and management across countries. Japan and the United States offer contrasting approaches to addressing this issue, presenting a valuable opportunity for comparative analysis. Objective: This review examines the effectiveness of public health policies, cultural dietary habits, and lifestyle factors in combating childhood obesity in Japan and the United States. It aims to identify actionable insights to inform global strategies for obesity prevention. Results: Japan exhibits one of the lowest childhood obesity rates globally, attributed to prevention-focused policies such as the food education program, stringent school lunch standards, and culturally ingrained healthy eating practices. These efforts are complemented by active lifestyle promotion through urban planning and school-based physical education programs. In contrast, the United States faces higher obesity rates due to systemic challenges, including socioeconomic disparities, reliance on processed foods, sedentary lifestyles, and inconsistent implementation of federal programs like the National School Lunch Program (NSLP) and Supplemental Nutrition Assistance Program Education (SNAP-Ed). Conclusions: This review highlights Japan’s success in aligning public health initiatives with cultural norms to achieve sustainable outcomes. In the United States, systemic barriers and cultural disconnects hinder obesity prevention efforts. Recommendations include adopting integrated, prevention-focused policies, addressing socioeconomic inequities, redesigning urban environments to promote active living, and fostering global collaboration. This comparative analysis underscores the importance of culturally tailored, multidimensional strategies for addressing childhood obesity and improving public health outcomes worldwide.

## 1. Introduction

Childhood obesity is a critical global public health issue that has reached epidemic proportions in recent decades [1]. In the United States, obesity rates among children and adolescents aged 2–19 years have risen significantly, increasing from 13.9% in 1999–2000 to 21.1% in 2021–2023 [2]. This upward trend poses serious public health challenges, necessitating urgent action to address the issue. Childhood obesity is strongly associated with an elevated risk of chronic conditions, including type 2 diabetes, cardiovascular diseases, hypertension, and certain cancers [3,4,5]. Additionally, it often leads to psychosocial challenges such as low self-esteem, depression, and social stigmatization, with effects that frequently persist into adulthood [6]. The economic burden of obesity is equally significant. According to Ward et al. (2021) [7], annual medical costs for adults with obesity are $1,861 higher per person than for those with a healthy weight, contributing to nearly $173 billion in medical expenditures. Additionally, according to a report released by Milken Institute (2016), the overall economic impact of obesity in the United States, including indirect costs like lost productivity, has been estimated to exceed $1.4 trillion annually (equivalent to 8.2 percent of U.S. GDP) [8]. 

Obesity is a multifactorial health condition arising from the interaction of individual behaviors and broader societal and environmental determinants [9,10]. Critical individual-level behaviors include dietary intake and physical activity, with diets high in caloric density, added sugars, and unhealthy fats combined with insufficient physical activity contributing significantly to obesity [11]. These behaviors, however, are deeply influenced by factors such as socioeconomic status, food accessibility, urban infrastructure, cultural practices, and public health policies. Addressing obesity, therefore, requires a multidimensional approach that accounts for these dynamic interactions.

Global disparities in childhood obesity rates [12] highlight the complex interplay of public health policies, cultural dietary habits, and lifestyle factors across different nations. For instance, Japan, for example, reports one of the lowest childhood obesity rates globally at 4.5%, despite being a highly industrialized and urbanized nation [13,14,15,16,17]. This success is mainly attributed to a cohesive approach that integrates prevention-focused policies, cultural dietary norms emphasizing moderation and variety, and initiatives programs and strict health policies. In contrast, the U.S. has a childhood obesity prevalence exceeding 20%, with rates driven by systemic inequities, increased availability of calorie-dense foods, and insufficient opportunities for physical activity [18]. While programs like the National School Lunch Program and SNAP-Ed aim to address these issues, they face challenges such as funding constraints, inconsistent implementation, and structural barriers like food deserts and socioeconomic disparities [19,20,21]. 

**Figure 1:**
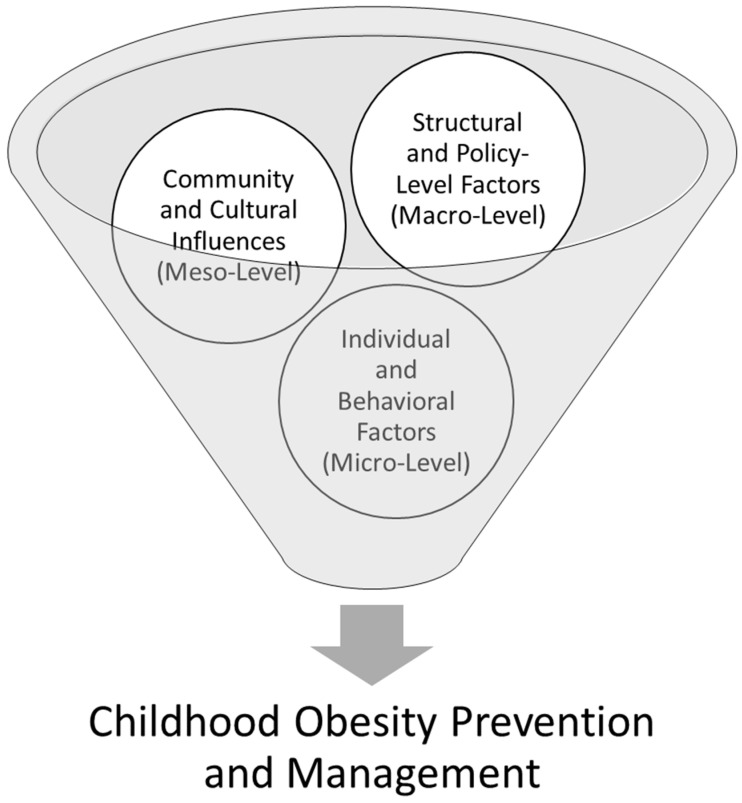
Interconnected factors influencing childhood obesity through the SEM.

Japan’s success in maintaining low obesity rates, despite the global upward trend, provides evidence-based insights into effective public health policies, cultural dietary habits, and lifestyle factors that could inform interventions in other countries like the USA, where childhood obesity rates remain alarmingly high. Guided by a framework derived from the social-ecological model (SEM) [22] (Figure 1), this review aimed to examine the effectiveness of public health policies, cultural dietary habits, and lifestyle factors in addressing childhood obesity in Japan and the United States. The SEM posits that health behaviors are shaped by interdependent influences across multiple levels, including Structural and Policy-Level Factors (Macro-Level), Community and Cultural Influences (Meso-Level), and Individual and Behavioral Factors (Micro-Level) [23]. Specifically, this review discussed the comparative effectiveness of public health policies, cultural dietary habits, and lifestyle factors in shaping childhood obesity trends in Japan and the United States, focusing on how these elements interact within the framework of the SEM to influence health behaviors and outcomes across individual, interpersonal, community, and policy levels. By addressing these levels of intervention, we ultimately aim to provide an actionable framework and evidence-based recommendations for policymakers, educators, and healthcare professionals to design culturally tailored, multidimensional strategies that effectively combat childhood obesity not only in the US but also on a global scale.

## 2. Results

### 2.1. Structural and Policy-Level Factors (Macro-Level)

#### 2.1.1. Japan

Japan’s public health policies embody a comprehensive, multi-level approach, closely aligned with the SEM framework, addressing childhood obesity across individual, organizational, and policy levels. Rooted in prevention and education, these policies focus on cultivating lifelong healthy habits, beginning in early childhood [24]. This holistic strategy integrates efforts at the school, family, and community levels, creating a cohesive framework that encourages health-promoting behaviors across all facets of society.

A cornerstone of Japan’s strategy is the *Shokuiku* program “food education” institutionalized under the Basic Law on *Shokuiku* enacted in 2005. This program prioritizes nutritional literacy, emphasizing the importance of balanced diets, proper eating behaviors, and the cultural value of food in promoting physical and mental well-being [16]. At the national level, *Shokuiku* establishes standardized guidelines for nutrition education, ensuring consistency across the country. Schools play a pivotal role in this framework by incorporating food education into their curricula, reinforced by the *Shokuiku* School Lunch Act [25]. This legislation mandates that school meals meet strict nutritional standards and utilize fresh, locally sourced ingredients, a policy that sets Japan apart from many other nations. Unlike the outsourcing commonly seen in many Western countries like the US, meals in Japanese schools are prepared in-house, ensuring high-quality nutrition. Students actively participate in serving meals and cleaning up afterward, fostering a sense of responsibility and respect for food [26]. This system not only ensures that children receive at least one nutritious meal daily but also instills lifelong habits of healthy eating. By integrating *Shokuiku* into the school curriculum, children are taught about various aspects of food, including its origins, preparation methods, and the cultural significance of traditional Japanese meals [26]. The program extends beyond schools, with family involvement being a key component. Parents are encouraged to adopt healthy meal planning practices, supported by workshops and community campaigns designed to reinforce the principles of *Shokuiku* at home. This integration of food education into daily life ensures that lessons learned in the classroom translate into lasting behavioral changes. Cultural traditions further support these efforts, promoting individual habits such as portion control and mindful eating, which are reinforced through both educational systems and community initiatives.

A unique feature of Japan’s public health strategy is its system of mandatory annual health check-ups for schoolchildren. These examinations monitor weight, height, BMI, and other key health indicators, enabling early detection of potential health issues, including obesity [27]. This system not only supports timely interventions but also informs national health policies by providing valuable data on trends and outcomes. Parents are actively involved in the process, receiving detailed health reports and personalized guidance to encourage healthier behaviors at home [28]. By addressing childhood obesity through aligned efforts at the policy, organizational, interpersonal, and individual levels, Japan demonstrates the efficacy of a cohesive, prevention-oriented approach.

#### 2.1.2. USA

In contrast to Japan’s proactive and unified strategy, public health policies in the U.S. often focus on reactive measures, addressing the consequences of childhood obesity rather than its root causes. Federal and state-level programs in the U.S. tend to operate in silos, frequently struggling to achieve the multi-level integration envisioned by the SEM. This fragmentation results in interventions that lack the alignment and consistency seen in Japan.

One of the most significant federal programs is the National School Lunch Program (NSLP), which serves over 29 million children daily [21,29]. The NSLP aims to provide nutritious meals to children, particularly those from low-income families, and was bolstered by the Healthy, Hunger-Free Kids Act of 2010. This legislation sought to align school meals with dietary recommendations by increasing the inclusion of whole grains, fruits, vegetables, and lean proteins, while reducing sodium, sugar, and unhealthy fats [30]. However, while the program is beneficial in addressing food insecurity though improving access to healthy food for children [31], it has faced criticism for inconsistent nutritional standards and challenges in implementation, limiting its effectiveness. For example, schools serve as key platforms for intervention, yet many lack the infrastructure, staffing shortages, or funding to meet updated dietary standards [21]. Another significant issue lies in the program’s reliance on processed and prepackaged foods, which are easier to prepare and distribute but often fall short in providing optimal nutritional value. For instance, while schools are required to meet specific calorie and nutrient requirements, the widespread use of processed foods frequently leads to higher levels of added sugars and preservatives, undermining the program’s intent [32]. This reliance also exacerbates disparities in meal quality between wealthier and under-resourced school districts, with the latter often unable to afford fresh, locally sourced ingredients [33]. Moreover, variations in meal quality and implementation fidelity between districts highlight systemic inequities. Wealthier districts with better funding and infrastructure are often better equipped to meet and exceed nutritional standards, providing students with more appealing and healthier meals. Conversely, schools in low-income areas may struggle to offer meals that are both nutritionally adequate and palatable, leading to issues such as food waste and reduced participation rates among students [34]. These challenges are compounded by the lack of consistent monitoring and enforcement mechanisms to ensure compliance with nutritional guidelines. Many schools report difficulty balancing cost-effectiveness with nutritional goals, and the program’s reimbursement rates often fail to cover the full cost of preparing healthier meals, leaving districts to absorb the shortfall [21,35]. This financial pressure discourages innovation and limits schools’ ability to offer diverse and high-quality food options.

The Supplemental Nutrition Assistance Program Education (SNAP-Ed) complements the NSLP by focusing on educating low-income families about nutrition, meal planning, and healthy eating [36]. This program was designed to empower individuals and families by providing education on nutrition, meal planning, and healthy eating habits. Through a combination of community-based programs, workshops, and educational campaigns, SNAP-Ed encourages participants to make healthier food choices and maximize the nutritional value of their resources [36]. Despite its potential benefits, the program’s impact is often constrained by systemic barriers that disproportionately affect low-income communities. One of the most pressing challenges is the prevalence of food deserts (geographic areas where access to affordable, nutritious food is severely limited due to a lack of grocery stores or reliable transportation options) [37,38]. In these areas, families often rely on convenience stores or fast-food outlets, which typically offer calorie-dense, nutrient-poor options. This reality undermines SNAP-Ed’s goals, as the lack of access to fresh fruits, vegetables, and whole grains significantly restricts families’ ability to implement the program’s dietary recommendations [38,39]. Moreover, SNAP-Ed’s effectiveness is further hindered by inconsistent funding and variations in program implementation across states. While some states have developed innovative community gardens and farmers’ market initiatives to address local needs, others face resource constraints that limit the reach and scope of their programs [40]. Additionally, educational materials and workshops may not fully account for cultural and linguistic diversity, reducing their relevance and appeal to certain populations [41]. Another barrier is the overarching issue of time and convenience for low-income families. Many participants face demanding work schedules and limited kitchen facilities, making it difficult to prepare meals from scratch, even if healthier food options are available [42,43]. These constraints highlight the need for SNAP-Ed to integrate practical solutions, such as promoting affordable pre-prepared healthy meal kits or collaborating with local businesses to expand access to fresh produce.

Furthermore, Let’s Move! Campaign was initiated in 2010 by former First Lady Michelle Obama, aimed at addressing childhood obesity through a multifaceted approach targeting improved school meal standards, increased physical activity opportunities, and healthier food marketing practices [44]. One of its key components, Let’s Move! Active Schools (LMAS), emphasized enhancing physical activity opportunities in schools. Evaluations of LMAS initiatives, such as grants provided by ChildObesity180 and Fuel Up to Play 60, demonstrated statistically significant improvements in implementing school-level physical education (PE) and physical activity (PA) practices. These included promoting PA through messaging, introducing classroom PA breaks, and offering PA before and after school [45,46]. Overall, the campaign successfully raised national awareness and facilitated meaningful policy and practice changes, including updates to the National School Lunch Program (NSLP) and increased adoption of PA guidelines in schools. However, its long-term impact has been questioned due to systemic challenges such as inconsistent implementation, insufficient sustainable funding, and limited follow-through on key initiatives. Additionally, the lack of robust mechanisms to ensure policy continuity has hindered its ability to achieve lasting outcomes [45,47].

Overall, the US faces significant challenges in aligning public health policies with the multi-level framework required for sustained impact. Systemic inequities, inconsistent funding, and cultural barriers create a fragmented landscape that struggles to achieve the cohesion seen in Japan. Addressing these issues will require a shift toward prevention-focused policies that integrate efforts across federal, state, and local levels while addressing the root causes of childhood obesity at both structural and cultural levels.

### 2.2. Community and Cultural Influences (Meso-Level)

#### 2.2.1. Japan

Cultural dietary habits in Japan are deeply intertwined with the nation’s history, traditions, and values, playing a significant role in maintaining its low rates of childhood obesity. The traditional Japanese diet, known as *Washoku*, exemplifies balance, variety, and moderation. This dietary pattern, which is recognized by UNESCO as an Intangible cultural heritage, prioritizes fresh, minimally processed foods such as vegetables, fish, rice, soy products, and fermented items like miso and pickles [48]. These components are not only rich in essential nutrients but also low in saturated fats and high in fiber, supporting metabolic health and effective weight management [49,50]. The nutritional integrity of *Washoku* is enhanced by the careful preparation and mindful consumption of meals, which emphasizes small portions served in individual dishes. This practice promotes slower eating and greater awareness of satiety cues, reducing the likelihood of overeating, a stark contrast to the larger portion sizes prevalent in Western cultures [51]. 

In Japanese culture, family mealtimes are highly valued as opportunities for nourishment, education, and socialization. Parents and caregivers model healthy eating behaviors, encouraging children to explore a variety of foods and appreciate their flavors and textures. This cultural emphasis on communal meals reinforces positive eating habits while allowing parents to monitor their children’s dietary intake [52,53]. The concept of seasonality further shapes Japanese dietary habits, with meals reflecting the availability of fresh, seasonal ingredients. Eating in harmony with nature ensures dietary variety while promoting the consumption of foods at their nutritional peak. Traditional food preparation methods, such as steaming, grilling, and fermenting, preserve the natural nutrients of ingredients while enhancing their flavors [53]. 

Cultural practices such as *ichiju-sansai* (one soup, three sides) ensure nutritionally balanced meals that combine carbohydrates, proteins, and vegetables. Schools also play a crucial role in reinforcing these habits by designing lunch programs that adhere to national nutritional guidelines. These programs educate children about the importance of nutrition and the cultural significance of food, fostering lifelong healthy eating behaviors. Through this seamless integration of cultural traditions and public health initiatives, Japan has cultivated a societal framework that not only supports healthy eating but also minimizes childhood obesity. The mindfulness around food and the communal emphasis in Japanese culture provide an exemplary model for global health promotion [53].

#### 2.2.2. USA

In contrast, the dietary habits in the U.S. are shaped by convenience, aggressive marketing, and systemic socioeconomic factors, resulting in significant challenges to addressing childhood obesity. The Standard American Diet (SAD) is characterized by the high consumption of processed and ultra-processed foods loaded with added sugars, unhealthy fats, and sodium [54,55]. These types of food, which are often aggressively marketed to children through colorful packaging and media advertisements, significantly contribute to excessive caloric intake and poor nutritional quality, fueling the rising prevalence of diet-related chronic conditions such as obesity, type 2 diabetes, and cardiovascular disease [56,57,58]. 

A defining feature of the American dietary landscape is its reliance on fast food and prepackaged convenience items. These calorie-dense but nutritionally imbalanced options are often the default for families facing time constraints, limited cooking skills, and economic pressures [59]. This dependency is exacerbated by aggressive marketing strategies that target children with brightly colored packaging and enticing advertisements, fostering unhealthy eating behaviors from an early age [60]. Additionally, the normalization of large portion sizes—both in restaurants and at home—further compounds the issue. Many meals in the U.S. significantly exceed recommended serving sizes, distorting perceptions of appropriate food quantities and contributing to habitual overeating [61,62]. This normalization of oversized portions distorts perceptions of appropriate food quantities, particularly among children, and contributes to overeating [63,64]. 

The abundance of inexpensive, energy-dense foods disproportionately affects low-income communities, where access to fresh produce and healthier alternatives is often limited. These systemic barriers are exacerbated by the existence of food deserts, and geographic areas lacking affordable and nutritious food options [65,66,67]. Efforts to improve dietary habits, such as incorporating healthier options into school lunch programs and implementing community-based initiatives, have shown promise but remain insufficient to counteract deeply rooted cultural preferences for convenience-driven eating habits. Moreover, the aggressive marketing of ultra-processed foods continues to undermine these efforts, particularly in vulnerable populations [68].

Overall, the differences between Japan’s mindful, balanced dietary practices and the U.S.’s convenience-driven approach highlight the significant cultural and systemic factors contributing to childhood obesity. Japan’s emphasis on tradition, mindfulness, and nutrition education offers valuable lessons for promoting healthy behaviors through culturally aligned strategies. Conversely, addressing the challenges in the U.S. will require coordinated efforts to tackle systemic inequities, expand access to nutritious food, and shift cultural norms around diet and lifestyle. Without these systemic changes, efforts to combat childhood obesity in the U.S. will continue to face significant obstacles.

### 2.3. Individual and Behavioral Factors (Micro-Level)

#### 2.3.1. Japan

Physical activity is another pillar of Japan’s public health strategy, deeply embedded in cultural, structural, and societal norms [69]. Japanese children benefit from an environment that actively promotes movement, with daily routines designed to encourage physical activity. One prominent example is walking or cycling to school, which is a common practice supported by urban planning that prioritizes pedestrian and bicycle-friendly infrastructure [70]. Each municipality’s board of education ensures that school commuting methods are safe and feasible, considering local geography, climate, and transportation challenges. In urban areas, where schools are often within walking distance of children’s homes, walking is the preferred method of commuting. Safety measures, such as parent and community volunteer supervision, ensure these routines remain secure and accessible. This daily practice not only fosters regular physical activity but also reflects broader societal efforts to embed active living into everyday life through collaborative, localized initiatives [71].

Schools play a pivotal role in reinforcing physical activity through mandatory physical education (PE) programs. These programs are not solely focused on developing motor skills and physical fitness but also emphasize teamwork, discipline, and fostering a lifelong appreciation for active lifestyles [28,72]. Beyond traditional PE classes, Japanese schools incorporate physical activity into daily routines through practices such as “Rajio Taiso” (radio calisthenics), which involves light, structured exercises performed every morning. These routines prepare students for the day ahead while promoting the habit of consistent movement [72,73]. Additionally, extracurricular sports clubs are also an integral part of school life providing students with opportunities to specialize in various activities and compete at local and national levels, further reinforcing the value of regular exercise. Beyond these sessions, schools often organize extracurricular sports programs and seasonal events, such as sports days (*undokai*), that involve both students and their families [74]. These activities not only promote physical health but also reinforce a sense of community and collective responsibility for well-being.

Beyond the school environment, leisure time in Japan often revolves around outdoor activities, shaped by a cultural identity that values nature and communal engagement [75,76]. Public spaces such as parks and recreational areas are widely available and frequently utilized by families. Japan’s local governments and communities actively promote physical activity through organized sports events, recreational programs, and accessible public spaces. Initiatives such as weekend sports festivals, park fitness classes, and community athletic leagues aim to involve children and their families, creating a culture of active living that extends beyond childhood [28,71]. Furthermore, traditional cultural practices, such as martial arts or “*Budo*” (e.g., judo, kendo), offer additional opportunities for physical activity and contribute to the holistic development of children [77,78]. Although Japan has traditionally emphasized outdoor play and social activities, modern trends indicate a rise in screen time among children. Despite efforts by parents and schools to regulate device usage, approximately 50% of Japanese children now exceed the recommended two hours of daily screen time [79]. This shift presents a growing challenge, as excessive screen time is associated with sedentary behaviors that increase the risk of obesity and other health issues. Nonetheless, many Japanese families strive to balance technology use with physical activities, reflecting an ongoing effort to uphold traditional values in the face of modern influences.

#### 2.3.2. USA

In contrast, children’s lifestyles in the U.S. are increasingly defined by sedentary behaviors, influenced by technological advancements, cultural norms, and urban design limitations. On average, children and adolescents in the U.S. spend 6 to 8 h per day engaged in sedentary activities, both during and outside of school [80,81].

The urban design in many parts of the U.S. limits opportunities for physical activity. Suburban trails and car-centric infrastructure often make active transportation, such as walking or biking to school, impractical or unsafe. According to data from the National Center for Safe Routes to School (2023) [82,83], less than 15% of children actively commute (walk or bike) to school, compared to nearly 50% in the 1960s. This decline is attributed to factors such as urban sprawl, increased reliance on motor vehicles, and concerns about traffic safety. The lack of infrastructure to support active commuting, such as sidewalks, bike lanes, and safe crossing zones, further discourages walking or biking [82,83]. Parents’ concerns about neighborhood safety and the distance between homes and schools also play a role in this trend [84]. As a result, the opportunity for children to integrate physical activity into their daily routines is significantly reduced, exacerbating the sedentary lifestyles associated with childhood obesity.

Neighborhood design further restricts opportunities for physical activity, particularly in low-income areas where sidewalks, parks, and recreational facilities are either inadequate or entirely absent [85,86]. American urban planning often discourages incidental exercise, such as walking short distances or using public transportation. This stands in stark contrast to more pedestrian-friendly cities, where urban design encourages incidental exercise through walking and public transportation use [87]. Environmental factors also play a crucial role in shaping children’s activity levels. The community neighborhood space environment has been identified as critical for promoting outdoor activity among children, yet in many urban areas, these spaces fall short of providing accessible and safe environments for play [88]. However, in many U.S. urban areas, for example, the design of these spaces often falls short of promoting active lifestyles.

Physical activity policies in the U.S. vary significantly by state and school district, further complicating efforts to promote active lifestyles among children. While most schools are required to include physical education (PE) in their curriculum, the quality, duration, and frequency of PE programs differ widely. Budget constraints and competing academic priorities often result in reduced physical activity (PA) opportunities within the school day. Studies indicate that only a minority of schools meet recommended daily PE guidelines, with many prioritizing academic instructions over physical activity [89,90,91]. Furthermore, disparities in funding and resources also can contribute to variability in program implementation, leaving low-income schools particularly disadvantaged. Additionally, access to extracurricular sports and fitness programs is often inequitable, as participation is frequently tied to household income and community resources. Children from low-income families may face financial barriers, such as fees for participation, equipment, or transportation.

Beyond physical activity, technology and screen time, including smartphones, tablets, and gaming consoles, also play a significant role in shaping the sedentary behaviors of American children. Recent data indicate that American children spend an average of over 7 h per day engaged in screen-based activities, far surpassing the American Academy of Pediatrics’ recommended limit of 2 h per day for entertainment-related screen time [92,93]. Prolonged screen exposure not only displaces time that could be spent engaging in physical activity but also promotes sedentary behavior, a known risk factor for obesity. Additionally, excessive screen use is associated with increased consumption of high-calorie, nutrient-poor snacks during screen time, further compounding the risk of unhealthy weight gain and other adverse health outcomes. Addressing these trends will require coordinated efforts to reduce screen time, improve access to recreational spaces, and encourage active lifestyles across all socioeconomic groups.

### 2.4. Interplay of Factors

Childhood obesity is a complex, multifactorial condition influenced by the interplay of individual behaviors, community norms, and structural policies. These factors do not operate in isolation but interact dynamically to shape dietary habits, physical activity levels, and overall health outcomes. At the individual level, children’s food choices, physical activity, and screen time habits directly impact obesity risk. However, these behaviors are shaped by community influences, including cultural norms, food availability, and access to safe recreational spaces. Communities that promote balanced diets, active commuting, and structured physical education foster healthier behaviors, while those characterized by processed food consumption and sedentary lifestyles contribute to obesity.

Structural policies set the foundation for these environments. Government regulations on school meals, public health campaigns, and urban planning shape food accessibility and physical activity opportunities. Countries with cohesive policies, such as Japan’s *Shokuiku* initiative, ensure consistent nutritional education and support active lifestyles, reducing obesity prevalence. In contrast, fragmented policies, food deserts, and socioeconomic disparities in healthcare access, as seen in many Western nations, exacerbate obesity rates, particularly in vulnerable populations.

These levels are deeply interconnected. Effective policies shape healthier communities, reinforcing positive individual behaviors. When policies fail to support healthy environments, obesogenic conditions emerge, making it harder for individuals to maintain healthy habits. This comparative analysis, summarized in Table 1, highlights the necessity of a tailored, multi-level approach that aligns personal choices, community resources, and policy-driven interventions to create sustainable, health-promoting environments.

### 2.5. Global Implications

The comparison between Japan and the US highlights the critical importance of context-specific strategies in tackling childhood obesity. Japan’s model underscores the effectiveness of prevention-focused, culturally aligned policies that integrate seamlessly into societal norms, creating a sustainable framework for promoting healthy behaviors. In contrast, the challenges faced by the U.S. illustrate the complexities of addressing systemic inequities and cultural disconnects within diverse and heterogeneous populations. These contrasting approaches provide valuable insights for global public health, emphasizing that successful interventions must be tailored to the unique cultural, economic, and structural contexts of each setting.

This comparison reinforces the need for multidimensional strategies that address both individual behaviors and broader societal determinants. Countries aiming to combat childhood obesity should consider not only implementing policies but also ensuring their alignment with cultural practices and structural realities. By adopting such tailored approaches, public health initiatives can achieve long-term sustainability and effectiveness in diverse global contexts.

## 3. Recommendations

Addressing childhood obesity requires a comprehensive, evidence-based approach that integrates effective policies, cultural values, and supportive environments. Insights from the comparative analysis of Japan and the U.S. offer actionable strategies for enhancing obesity prevention efforts.

### 3.1. Adopt Integrated, Prevention-Focused Policies

Japan’s prevention-focused public health framework, exemplified by the *Shokuiku* program, underscores the importance of integrating nutrition education and physical activity into school systems, families, and communities. While school-based interventions have been widely implemented, their effectiveness in reducing obesity has yielded mixed results. Large-scale studies such as the HEALTHY Study [94] and the Pathways Study [95] suggest that while these programs can improve dietary behaviors, physical activity levels, and metabolic markers, they may not always lead to significant reductions in BMI or adiposity. These findings highlight the complexity of behavior change and the need for multilevel approaches that extend beyond schools.

Rather than viewing school-based interventions as standalone solutions [96,97], the U.S. could benefit from embedding them within a broader public health strategy that includes family engagement, community-based initiatives, and policy-driven environmental changes. Aligning nationwide nutrition education with standardized physical activity curricula, as seen in Japan, would help establish foundational health behaviors early in life. Additionally, a cohesive national strategy coordinating efforts across federal, state, and local levels could enhance program consistency and equity. Implementing a system of regular health monitoring, akin to Japan’s mandatory school health check-ups, could facilitate early detection of health concerns and timely, targeted interventions.

### 3.2. Leverage Cultural Values to Promote Healthy Eating

Cultural values play a critical role in shaping dietary behaviors. Japan’s emphasis on portion control, communal meals, and seasonal eating reflects an alignment with health-conscious norms. In contrast, the U.S. faces challenges rooted in fast food culture, processed meals, and oversized portions. Public health initiatives in the U.S. should work to reshape cultural attitudes by promoting mindful eating, smaller portion sizes, and the benefits of family meals. Behavioral insights can guide campaigns and educational programs that encourage nutritional balance and moderation. By integrating these principles into schools and community programs, long-term cultural shifts toward healthier eating habits can be achieved.

### 3.3. Prioritize Urban Planning and Infrastructure for Active Lifestyles

Physical activity is a key determinant of childhood obesity, and Japan’s pedestrian-friendly urban design, coupled with cultural norms limiting screen time, supports active lifestyles. In contrast, systemic barriers in the United States, such as suburban sprawl, car-centric infrastructure, and limited recreational spaces, restrict opportunities for physical activity. Policymakers should prioritize urban planning initiatives that create walkable neighborhoods, safe cycling routes, and accessible parks. Investments in school facilities, such as playgrounds and sports complexes, would provide equitable access to physical activity for children across socioeconomic backgrounds. Programs like Safe Routes to School could further encourage active commuting and foster long-term engagement in physical activity.

### 3.4. Address Socioeconomic Inequities and Food Accessibility

Socioeconomic disparities and food deserts present significant barriers to obesity prevention in the United States. Low-income families often rely on calorie-dense, nutrient-poor foods due to affordability and accessibility challenges. Targeted interventions, such as subsidies for fresh produce, tax incentives for grocery stores in underserved areas, and the expansion of programs like SNAP-Ed, could improve food accessibility and affordability. Japan’s emphasis on equity through community-driven programs and government-funded initiatives serves as a model for bridging these gaps. Enhancing food accessibility would address the root causes of obesity while fostering overall nutritional equity.

### 3.5. Foster Public–Private Partnerships for Sustainable Change

Public–private collaborations can drive systemic changes by aligning public health goals with market-driven incentives. For example, food manufacturers could be incentivized to reformulate products, reducing added sugar and sodium. Schools and community organizations could partner with local businesses to provide affordable, nutritious meals. These partnerships would create a sustainable ecosystem that supports healthier food environments and lifestyle choices, fostering widespread adoption of health-conscious behaviors.

### 3.6. Promote Cross-Cultural Learning and Global Collaboration

Global collaboration offers valuable opportunities to combat childhood obesity by sharing knowledge and best practices. Japan’s success in school-based nutrition education and the United States’ innovative approaches to community engagement and technological solutions can serve as mutual learning opportunities. International forums, such as the World Health Organization (WHO) and the United Nations Children’s Fund (UNICEF), can facilitate research partnerships, capacity-building initiatives, and knowledge exchange. These collaborations could accelerate the development of effective, context-sensitive interventions on a global scale.

### 3.7. Incorporate Technological Innovations in Obesity Prevention

Emerging technologies, including wearable devices, virtual reality (VR), and mobile health applications, hold transformative potential in obesity prevention. These tools can provide personalized dietary guidance, promote physical activity, and engage families through interactive educational programs. The United States, with its advanced technological infrastructure, is well-positioned to lead in developing and implementing such innovations. Collaborations with Japan, known for its effective integration of technology in education, could amplify these efforts. Pilot programs leveraging technology could inform scalable, data-driven solutions tailored to diverse populations.

## 4. Conclusions

Childhood obesity is a complex and pressing global public health challenge that demands holistic, culturally sensitive, and multidimensional solutions. This review examined the effectiveness of public health policies, cultural dietary habits, and lifestyle factors in addressing childhood obesity in Japan and the United States. Japan’s success lies in its prevention-focused, culturally integrated approach, exemplified by *Shokuiku*, mindful eating traditions, and strong support for physical activity. In contrast, the U.S. faces systemic challenges, including socioeconomic disparities, the dominance of processed foods, and urban environments that limit active living, which weaken obesity prevention efforts.

The findings from this review underscore the critical need for integrated strategies that go beyond addressing individual behaviors, instead targeting the broader structural and societal determinants of obesity. Japan provides a compelling model for early prevention, nutritional education, and community engagement, while the U.S. offers insights into the role of technology, policy adaptation, and social programs in tackling health disparities. Both nations have much to learn from one another, reinforcing the importance of tailoring interventions to cultural, economic, and structural contexts to maximize impact.

Ultimately, combating childhood obesity requires global collaboration, where nations share best practices, foster cross-cultural learning, and implement evidence-based strategies that account for unique societal factors. By taking a context-driven, multidimensional approach, countries can develop sustainable solutions to improve children’s health outcomes worldwide. The lessons drawn from this comparative analysis provide a foundation for more effective interventions, ensuring that future generations grow up in environments that support healthy eating, active living, and long-term well-being.

## Figures and Tables

**Table 1:** Comparative overview of childhood obesity strategies: insights from Japan and the United States.

	Japan	United States
Structural and Policy-Level Factors (Macro-Level)	Prevention-focused, holistic approach; includes *Shokuiku* (food education), strict school lunch standards, mandatory physical education, and annual health check-ups	Reactive approach with programs like the NSLP and SNAP-Ed, which aim to reduce disparities but face funding and implementation challenges
Community and Cultural Influences (Meso-Level)	Traditional diet (e.g., *washoku*) emphasizes portion control, communal eating, fresh seasonal foods, and nutritional balance; promotes mindful eating.	Diet characterized by high consumption of processed, calorie-dense foods, large portion sizes, heavy reliance on fast food, and aggressive marketing targeted at children.
Individual and Behavioral Factors (Micro-Level)	Active commuting to school, structured physical education programs, regular outdoor activities, and lower screen time; urban and cultural designs promote active living.	Predominantly sedentary lifestyles with limited active commuting, variable physical education quality, high screen time, and insufficient infrastructure for recreational physical activity.
Structural Challenges	Minimal disparities in access to nutrition and activity resources; policies emphasize equity through public funding and community involvement	Significant socioeconomic disparities, the prevalence of food deserts, and limited recreational spaces disproportionately affect low-income families and under-resourced schools.
Recommendations	Strengthen prevention strategies, enhance monitoring systems, and proactively address emerging challenges like urbanization and increased screen time.	Improve coordination across federal, state, and local levels; enhance access to nutritious foods, redesign urban planning for active living, and regulate food marketing to children.
Global Implications	Provides a model for prevention-focused strategies that align cultural values with public health goals, offering lessons for countries aiming to address obesity proactively.	Illustrate challenges in addressing socioeconomic disparities and aligning public health interventions in diverse populations with varying access to resources and infrastructure.

## References

[1] Di Cesare M., Sorić M., Bovet P., Miranda J.J., Bhutta Z., Stevens G.A., Laxmaiah A., Kengne A.-P., Bentham J. (2019). The epidemiological burden of obesity in childhood: A worldwide epidemic requiring urgent action. BMC Med..

[2] Emmerich S., Ogden C. (2024). Prevalence of Obesity and Severe Obesity Among Persons Aged 2–19 Years—United States, 1999–2000 Through 2021–2023. MMWR Morb. Mortal. Wkly. Rep..

[3] Chung S.T., Krenek A., Magge S.N. (2023). Childhood Obesity and Cardiovascular Disease Risk. Curr. Atheroscler. Rep..

[4] Balasundaram P., Krishna S. (2023). Obesity Effects on Child Health. [Updated 2023 Apr 10]. StatPearls [Internet].

[5] Kansra A.R., Lakkunarajah S., Jay M.S. (2020). Childhood and Adolescent Obesity: A Review. Front. Pediatr..

[6] Rankin J., Matthews L., Cobley S., Han A., Sanders R., Wiltshire H.D., Baker J.S. (2016). Psychological consequences of childhood obesity: Psychiatric comorbidity and prevention. Adolesc. Health Med. Ther..

[7] Ward Z.J., Bleich S.N., Long M.W., Gortmaker S.L. (2021). Association of body mass index with health care expenditures in the United States by age and sex. PLoS ONE.

[8] Milken Institute (2016). Americans’ Obesity Weighs Down U.S. Economy by $1.4 Trillion|Milken Institute. Milken Institute. https://milkeninstitute.org/content-hub/news-releases/americans-obesity-weighs-down-us-economy-14-trillion.

[9] De Lorenzo A., Romano L., Di Renzo L., Di Lorenzo N., Cenname G., Gualtieri P. (2020). Obesity: A preventable, treatable, but relapsing disease. Nutrition.

[10] Lee A., Cardel M., Donahoo W.T. (2019). Social and environmental factors influencing obesity. Endotext [Internet].

[11] Moschonis G., Trakman G.L. (2023). Overweight and obesity: The interplay of eating habits and physical activity. Nutrients.

[12] Obita G., Alkhatib A. (2022). Disparities in the Prevalence of Childhood Obesity-Related Comorbidities: A Systematic Review. Front. Public Health.

[13] Japan National Health and Nutrition 2019 Survey (2019). Obesity Prevalence. https://www.e-stat.go.jp/stat-search/files?page=1&layout=datalist&toukei=00450171&tstat=000001041744&cycle=7&tclass1=000001148507&tclass2val=0.

[14] Lui D.T.W., Ako J., Dalal J., Fong A., Fujino M., Horton A., Krittayaphong R., Almahmeed W., Matthias A.T., Nelson A.J. (2024). Obesity in the Asia-Pacific region: Current perspectives. J. Asian Pac. Soc. Cardiol..

[15] Yamaguchi M., Nomura M., Arai Y., Vandevijvere S., Swinburn B., Nishi N. (2022). An assessment of implementation gaps and priority recommendations on food environment policies: The Healthy Food Environment Policy Index in Japan. Public Health Nutr..

[16] Miyoshi M., Tsuboyama-Kasaoka N., Nishi N. (2012). School-based “Shokuiku” program in Japan: Application to nutrition education in Asian countries. Asia Pac. J. Clin. Nutr..

[17] Kaneda M., Yamamoto S. (2015). The Japanese School Lunch and Its Contribution to Health. Nutr. Today.

[18] Stierman B., Afful J., Carroll M.D., Chen T.-C., Davy O., Fink S., Fryar C.D., Gu Q., Hales C.M., Hughes J.P. (2021). National Health and Nutrition Examination Survey 2017–March 2020 Prepandemic Data Files-Development of Files and Prevalence Estimates for Selected Health Outcomes. National Health Statistics Reports.

[19] USDA NIFA|National Institute of Food and Agriculture Supplemental Nutrition Education Program-Education (SNAP-Ed). https://www.nifa.usda.gov/grants/programs/capacity-grants/efnep/snap/supplemental-nutrition-education-program-education-snap-ed.

[20] Berkowitz S.A., Seligman H.K., Rigdon J., Meigs J.B., Basu S. (2017). Supplemental Nutrition Assistance Program (SNAP) participation and health care expenditures among low-income adults. JAMA Intern. Med..

[21] Toossi S., Todd J.E., Guthrie J., Ollinger M. (2008). The National School Lunch Program: Background, Trends, and Issues.

[22] Centers for Disease Control Prevention [CDC] (2015). The Social-Ecological Model: A Framework for Prevention.

[23] Wold B., Mittelmark M.B. (2018). Health-promotion research over three decades: The social-ecological model and challenges in implementation of interventions. Scand. J. Public. Health.

[24] Kondo N. (2014). What Has Made Japan Healthy?—Contributions of local and governmental health policies. Jpn. Med. Assoc. J. JMAJ.

[25] Tanaka N., Miyoshi M. (2012). School lunch program for health promotion among children in Japan. Asia Pac. J. Clin. Nutr..

[26] Kurotani K., Shinsugi C., Miyoshi M., Takimoto H. (2020). Overviews of *Shokuiku* Promotion. Jpn. J. Nutr. Diet..

[27] Tomokawa S., Miyake K., Takahashi K., Tomokawa A., Kokudo S., Ueno M., Kigawa M., Asakura T. (2021). Health screening system to ensure children’s health and development in Japan. Pediatr. Int..

[28] Organisation for Economic Co-operation and Development (2019). OECD Reviews of Public Health. Japan: A Healthier Tomorrow.

[29] Newman C., Ralston K. (2006). Profiles of Participants in the National School Lunch Program: Data from Two National Surveys.

[30] U.S. Department of Agriculture (2017). FACT SHEET: Healthy, Hunger-Free Kids Act School Meals Implementation [Press Release].

[31] Kenney E.L., Barrett J.L., Bleich S.N., Ward Z.J., Cradock A.L., Gortmaker S.L. (2020). Impact Of The Healthy, Hunger-Free Kids Act On Obesity Trends. Health Aff..

[32] Gundersen C., Kreider B., Pepper J. (2012). The impact of the National School Lunch Program on child health: A nonparametric bounds analysis. J. Econom..

[33] Huang J., Barnidge E. (2016). Low-income Children’s participation in the National School Lunch Program and household food insufficiency. Soc. Sci. Med..

[34] Government Accountability Office (2014). School Lunch: Implementing Nutrition Changes Was Challenging and Clarification of Oversight Requirements Is Needed.

[35] Hirschman J., Chriqui J.F. (2013). School food and nutrition policy, monitoring and evaluation in the USA. Public Health Nutr..

[36] U.S. Department of Agriculture [USDA] (2024). SNAP-Ed. https://www.fns.usda.gov/snap/snap-ed.

[37] Beaulac J., Kristjansson E., Cummins S. (2009). Peer reviewed: A systematic review of food deserts, 1966–2007. Prev. Chronic Dis..

[38] Gearing M., Dixit-Joshi S., May L. (2021). Barriers that Constrain the Adequacy of Supplemental Nutrition Assistance Program (SNAP) Allotments: Survey Findings.

[39] Walker R.E., Keane C.R., Burke J.G. (2010). Disparities and access to healthy food in the United States: A review of food deserts literature. Health Place.

[40] Hilmers A., Hilmers D.C., Dave J. (2012). Neighborhood disparities in access to healthy foods and their effects on environmental justice. Am. J. Public Health.

[41] Leung C.W., Tester J.M. (2019). The Association between Food Insecurity and Diet Quality Varies by Race/Ethnicity: An Analysis of National Health and Nutrition Examination Survey 2011–2014 Results. J. Acad. Nutr. Diet..

[42] Jabs J., Devine C.M. (2006). Time scarcity and food choices: An overview. Appetite.

[43] Ravikumar D., Spyreli E., Woodside J., McKinley M., Kelly C. (2022). Parental perceptions of the food environment and their influence on food decisions among low-income families: A rapid review of qualitative evidence. BMC Public Health.

[44] Obama M. (2010). First Lady Michelle Obama Launches Let’s Move: America’s Move to Raise a Healthier Generation of Kids.

[45] Miller G.F., Sliwa S., Michael S., Lee S., Burgeson C., Krautheim A.M., Hatfield D.P., Sharma S., Economos C.D. (2018). Evaluation of Let’s Move! active schools activation grants. Prev. Med..

[46] Andersen J.A., Wylie L.E., Brank E.M. (2017). Public health framing and attribution: Analysis of the first lady’s remarks and news coverage on childhood obesity. Cogent Soc. Sci..

[47] Pilney S.J. (2024). Challenging the Let’s Move Campaign: Advocating for a Weight-Inclusive Approach to Public Health Programming. Ph.D. Thesis.

[48] Kumakura I., Ehara A., Okubo H. (2017). Washoku: Traditional Dietary Cultures of the Japanese.

[49] Gabriel A.S., Ninomiya K., Uneyama H. (2018). The Role of the Japanese Traditional Diet in Healthy and Sustainable Dietary Patterns around the World. Nutrients.

[50] Washoku World Challenge. What is Washoku?. https://www.washoku-worldchallenge.maff.go.jp/2020/en/learning/articles_01.html.

[51] Alldayieat (2023). Washoku Diet: Revitalize with Japanese Health. https://www.alldayieat.com/blog/washoku-diet/?utm_source=chatgpt.com.

[52] Adachi M. (2008). Theories of nutrition education and promotion in Japan: Enactment of the “Food Education Basic Law”. Asia Pac. J. Clin. Nutr..

[53] The Ministry of Agriculture, Forestry and Fisheries (MAFF) (2019). Current Dietary Situation in Japan and Promotion of Shokuiku (Food and Nutrition Education).

[54] Davis E. (2023). The Standard American Diet: What Is it and Where Do We Go Next?. https://www.doctorkiltz.com/standard-american-diet/.

[55] Clemente-Suárez V.J., Beltrán-Velasco A.I., Redondo-Flórez L., Martín-Rodríguez A., Tornero-Aguilera J.F. (2023). Global Impacts of Western Diet and Its Effects on Metabolism and Health: A Narrative Review. Nutrients.

[56] Smith R., Kelly B., Yeatman H., Boyland E. (2019). Food Marketing Influences Children’s Attitudes, Preferences and Consumption: A Systematic Critical Review. Nutrients.

[57] American Psychological Association (2013). The Impact of Food Advertising on Childhood Obesity.

[58] Coleman P.C., Hanson P., van Rens T., Oyebode O. (2022). A rapid review of the evidence for children’s TV and online advertisement restrictions to fight obesity. Prev. Med. Rep..

[59] Rahkovsky I., Jo Y., Carlson A. (2018). What Drives Consumers to Purchase Convenience Foods [Internet].

[60] Harris J.L., Graff S.K. (2011). Protecting Children From Harmful Food Marketing: Options for Local Government to Make a Difference. Prev. Chronic Dis..

[61] Spinella R. (2019). Portion Sizes in American Versus Other Countries. https://carolinianuncg.com/2019/08/28/portion-sizes-in-american-versus-other-countries/.

[62] Monteiro C.A., Cannon G. (2021). Yes, Food Portion Sizes and People Have Become Bigger and Bigger. What Is to Be Done?. Am. Public Health Assoc..

[63] Livingstone M.B.E., Pourshahidi L.K. (2014). Portion size and obesity. Adv. Nutr..

[64] Hetherington M.M., Blundell-Birtill P. (2018). The portion size effect and overconsumption–towards downsizing solutions for children and adolescents. Nutr. Bull..

[65] Larson N.I., Story M.T., Nelson M.C. (2009). Neighborhood environments: Disparities in access to healthy foods in the U.S. Am. J. Prev. Med..

[66] Sevilla N. (2021). Food Apartheid: Racialized Access to Healthy Affordable Food.

[67] Darmon N., Drewnowski A. (2015). Contribution of food prices and diet cost to socioeconomic disparities in diet quality and health: A systematic review and analysis. Nutr. Rev..

[68] Zhong A., Kenney E.L., Dai J., Soto M.J., Bleich S.N. (2022). The Marketing of Ultraprocessed Foods in a National Sample of U.S. Supermarket Circulars: A Pilot Study. AJPM Focus..

[69] Nagasawa T., Okuhara T., Terada M., Okada H., Goto E., Kiuchi T. (2023). Print Materials to Promote Physical Activities in Japan: Content Analysis from a Goal Theory. Healthcare.

[70] Waygood E.O.D., Taniguchi A. (2020). Japan: Maintaining high levels of walking. Transportation and Children’s Well-Being.

[71] Mori N., Armada F., Willcox D.C. (2012). Walking to school in Japan and childhood obesity prevention: New lessons from an old policy. Am. J. Public Health.

[72] Tanaka C., Abe T., Tanaka S., Hatamoto Y., Miyachi M., Inoue S., Reilly J.J. (2022). Results from the Japan 2022 report card on physical activity for children and youth. J. Exerc. Sci. Fit..

[73] Peterson H., Omizo N., Muronaka M. (2007). Adventures in Japanese 3: Textbook.

[74] Akiyama T. (2020). Undokai and sports events in the Japanese school system. Pediatr. Int..

[75] Leheny D.R. (2003). The Rules of Play: National Identity and the Shaping of Japanese Leisure.

[76] Varley P. (2000). Japanese Culture.

[77] Kowalczyk M., Zgorzalewicz-Stachowiak M., Błach W., Kostrzewa M. (2022). Principles of Judo Training as an Organised Form of Physical Activity for Children. Int. J. Environ. Res. Public Health.

[78] Verma S. (2024). Behavioral Improvement and Benefits of Traditional Martial Arts for Children Aged 7 to 12 Years. Int. J. Indian Psychȯl..

[79] Ishii K., Aoyagi K., Shibata A., Koohsari M.J., Carver A., Oka K. (2020). Joint Associations of Leisure Screen Time and Physical Activity with Academic Performance in a Sample of Japanese Children. Int. J. Environ. Res. Public Health.

[80] Matthews C.E., Chen K.Y., Freedson P.S., Buchowski M.S., Beech B.M., Pate R.R., Troiano R.P. (2008). Amount of time spent in sedentary behaviors in the United States, 2003–2004. Am. J. Epidemiol..

[81] Kohl H.W., Cook H.D. (2013). Status and Trends of Physical Activity Behaviors and Related School Policies, in Educating the Student Body: Taking Physical Activity and Physical Education to School.

[82] Safe Routes to Schools. https://www.saferoutespartnership.org/safe-routes-school.

[83] Etaati B. (2023). Understanding the Barriers to Active Transportation to School (ATS) and Evaluating the Impact of Educational Safety Programs on Traffic Behavior Trends in Students.

[84] (2023). Safe Routes Partnership. https://www.saferoutespartnership.org/.

[85] Gemmell E., Ramsden R., Brussoni M., Brauer M. (2023). Influence of Neighborhood Built Environments on the Outdoor Free Play of Young Children: A Systematic, Mixed-Studies Review and Thematic Synthesis. J. Urban Health.

[86] Parker L., Burns A.C., Sanchez E. (2009). Actions for Increasing Physical Activity, in Local Government Actions to Prevent Childhood Obesity.

[87] Young D.R., Cradock A.L., Eyler A.A., Fenton M., Pedroso M., Sallis J.F., Whitsel L.P., On behalf of the American Heart Association Advocacy Coordinating Committee (2020). Creating built environments that expand active transportation and active living across the United States: A policy statement from the American heart association. Circulation.

[88] Bao Y., Gao M., Luo D., Zhou X. (2021). Effects of Children’s Outdoor Physical Activity in the Urban Neighborhood Activity Space Environment. Front. Public Health.

[89] SHAPE America-Society of Health Physical Educators (2024). National Physical Education Standards.

[90] Lead A. (2021). Supporting Physical Education in Schools for All Youth.

[91] Sanchez-Vaznaugh E.V., Sánchez B.N., Rosas L.G., Baek J., Egerter S. (2012). Physical education policy compliance and children’s physical fitness. Am. J. Prev. Med..

[92] American College of Pediatricians (2020). Media Use and Screen Time—Its Impact on Children, Adolescents, and Families. https://acpeds.org/position-statements/media-use-and-screen-time-its-impact-on-children-adolescents-and-families.

[93] Paulus M.P., Squeglia L.M., Bagot K., Jacobus J., Kuplicki R., Breslin F.J., Bodurka J., Morris A.S., Thompson W.K., Bartsch H. (2019). Screen media activity and brain structure in youth: Evidence for diverse structural correlation networks from the ABCD study. Neuroimage.

[94] Foster G.D., Linder B., Baranowski T., Cooper D.M., Goldberg L., Harrell J.S., Kaufman F., Marcus M.D.,  Treviño R.P., HEALTHY Study Group (2010). A school-based intervention for diabetes risk reduction. N. Engl. J. Med..

[95] Caballero B., Clay T., Davis S.M., Ethelbah B., Rock B.H., Lohman T., Norman J., Story M., Stone E.J., Stephenson L. (2003). Pathways: A school-based, randomized controlled trial for the prevention of obesity in American Indian schoolchildren. Am. J. Clin. Nutr..

[96] Sallis J.F. (2018). Needs and Challenges Related to Multilevel Interventions: Physical Activity Examples. Health Educ. Behav..

[97] Sallis J.F., Cervero R.B., Ascher W., Henderson K.A., Kraft M.K., Kerr J. (2006). An ecological approach to creating active living communities. Annu. Rev. Public Health.

